# Material Transport Characteristics in Planetary Roller Melt Granulation

**DOI:** 10.3390/pharmaceutics15082039

**Published:** 2023-07-28

**Authors:** Tom Lang, Andreas Bramböck, Markus Thommes, Jens Bartsch

**Affiliations:** 1 Laboratory of Solids Process Engineering, Department of Biochemical and Chemical Engineering, TU Dortmund University, 44227 Dortmund, Germanyprofessors.fsv.bci@tu-dortmund.de (M.T.); 2 MeltPrep GmbH, 8020 Graz, Austria; andreas.bramboeck@meltprep.com

**Keywords:** continuous melt granulation, planetary roller granulator, residence time distribution

## Abstract

Melt granulation for improving material handling by modifying particle size distribution offers significant advantages compared to the standard methods of dry and wet granulation in dust reduction, obviating a subsequent drying step. Furthermore, current research in pharmaceutical technology aims for continuous methods, as these have an enhanced potential to reduce product quality fluctuations. Concerning both aspects, the use of a planetary roller granulator is consequential. The process control with these machines benefits from the enhanced ratio of heated surface to processed volume, compared to the usually-applied twin-screw systems. This is related to the unique concept of planetary spindles flowing around a central spindle in a roller cylinder. Herein, the movement pattern defines the transport characteristics, which determine the energy input and overall processing conditions. The aim of this study is to investigate the residence time distribution in planetary roller melt granulation (PRMG) as an indicator for the material transport. By altering feed rate and rotation speed, the fill level in the granulator is adjusted, which directly affects the average transport velocity and mixing volume. The two-compartment model was utilized to reflect these coherences, as the model parameters symbolize the sub-processes of axial material transport and mixing.

## 1. Introduction

Within the last decade, research in the field of pharmaceutical technology has focused on maximizing the potential of a systematic process design, following the Quality-by-Design approach as proclaimed in the ICH Q8 (R2) guideline [1] by establishing methods for continuous operation [2,3]. As well as a reduced machinery footprint, among others [4], a central advantage is the potential to reduce product quality fluctuations in comparison to batch processes. This is based on processing in a dynamic steady state, which is beneficial for implementing process control strategies [5], as here the central task is to maintain the process in balance.

**Figure 1:**
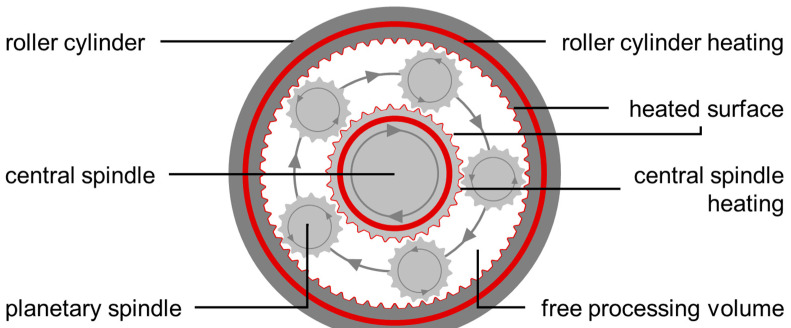
Radial cross-section of planetary roller processing section, including central spindle, planetary spindle, and roller cylinder. The heating of the roller cylinder and central spindle is independent. The central spindle, in addition to the number of planetary spindles, determines the free radial cross-section and free processing volume in axial direction.

This paradigm shift in the preferred manufacturing type also includes the unit operation granulation [6,7]. In comparison to dry [8,9] and wet granulation [10,11], as the most commonly applied methods in pharmaceutical technology, melt granulation offers the advantage of reduced fines and dust production, as well as the saving of a subsequent drying step [12]. This is especially valuable in terms of sustainability. In addition, this method enables the handling of moisture-sensitive materials [13] or the processing of high-dose formulations with poor compactibility [14]. To date, continuous melt granulation is typically executed on twin-screw machines [15,16,17]. However, the use of a planetary roller granulator, which belongs to the class of multiple screw machines which have already been applied for wet granulation [18,19,20], is a promising alternative [21] due to the basic process concept (Figure 1).

A firmly locked roller cylinder surrounds a motor-driven central spindle. Both parts have a 45° toothing and individual heating systems to regulate the energy input over the heated surface. The implemented planetary spindles determine the free processing volume. Within the processing section these are free-flowing and have to be distributed symmetrically across the radial cross-sectional area due to stability reasons. The minimum number is three in any case, while the maximum number depends on the apparatus size. During processing, the movement of the central spindle is superimposed onto the planetary spindles due to the toothing matching the other main parts. The resulting overall direction of circulation of the planetary spindles is concurrent to the central spindle, while its self-rotation is counter current. This radial movement pattern, in addition to the axial conveying related to the 45° toothing, defines the material transport characteristics during processing. The enhanced ratio of heated surface to free processing volume is beneficial in terms of temperature control. This is relevant for melt granulation, which is based on the softening or melting of the utilized binder component and the processing of temperature-sensitive materials.

During planetary roller melt granulation (PRMG), the product temperature and overall thermal stress correspond to the energy input, which is determined by the applied specific mechanical energy, the specific thermal energy, and the exposure time to the corresponding mechanisms. These can be characterized by the residence time distribution (RTD), an indirect process parameter frequently applied to evaluate and control various methods of pharmaceutical technology covering, e.g., mixing/blending [22,23] or tableting [24,25]. This, of course, also includes wet [26,27] or melt granulation [28] executed on twin-screw machines.

**Figure 2:**
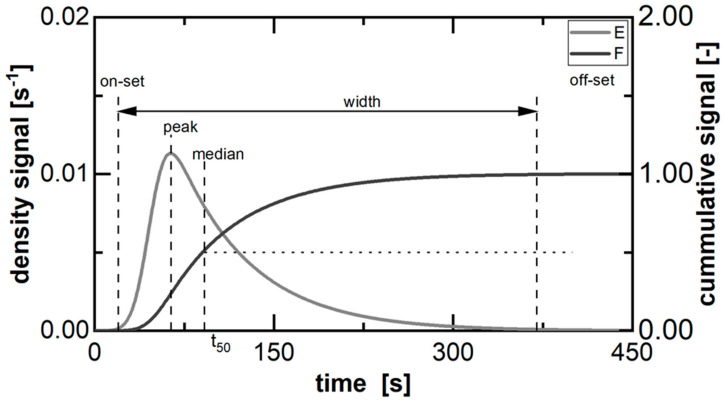
Typical profile of the residence time density function and corresponding cumulative form, including characteristic parameters.

The characteristics of an RTD (Figure 2) provide information on the sub-processes occurring during melt granulation, including the melting of the binder and the granulation mechanisms [29]. The on-set is the point in time of the first discharged material after entering the process at t = 0. Therefore, the time interval in between is the minimum duration of the material in the process. Thus, a temperature above the binder’s melting point has to be obtained in order to start the granulation. The off-set symbolizes the latest discharge of material after entering the process at t = 0. Therefore, the time interval in between is the maximum duration of applied shear and thermal stress to the material. This has to be considered in the context of breakage mechanisms during granulation [29] or thermal material degradation. The width as the time span between the on-set and off-set of the distribution reflects the overall back-mixing of the material during processing. This is crucial for the fundamental particle growth mechanisms [29]. Furthermore, the kinetic between peak and off-set is usually referred to as material “wash-out” and prolonged in comparison to the “loading” kinetic between on-set and peak. The median is representative for the overall RTD and average transport velocity.

The execution of a pulse experiment is the main procedure for determining the RTD in the form of the density function (*E*) [30,31,32]. Therefore, a tracer is added to the material stream at the inlet in order to measure the response signal in the outlet stream. This density function represents the probability of an individual entity exiting the process at a specific time (*t*). Via a step signal experiment, which utilizes a shift from a standard formulation to a tracer formulation or vice versa, the cumulative function (*F*) can be determined. This has a limit value of one (Equation (1)) as it is the sum function of the density distribution. In any case, a representative parameter for the RTD is the median (*t*_50_) representing the average transportation time (Equation (2)).

(1) $$F\;\left(t\to \infty \right)=\int_{0}^{t\to \infty }E(t)dt=1$$

(2) $$F\;\left(t_{50}\right)=\int_{0}^{t_{50}}E(t)dt=0.5.$$

The aim of this study is to identify the material transport and mixing characteristics during PRMG. Therefore, the RTD is applied as a key indicator for the processing conditions. In this respect, an on-line residence time determination method is evaluated in terms of repeatability as well as the suitability of a model approach from the literature to capture the experimental data. Furthermore, the impact of the process parameter variations on the machine fill level is discussed. These insights serve as basis for the characterization of the blending characteristics during processing, which refers to axial back-mixing or radial material distribution. Thus, the identified correlations between model parameters and processing conditions are highlighted.

## 2. Materials and Methods

### 2.1. Melt Granulation

Melt granulation was executed on a lab-scale planetary roller granulator (PWE 30, Entex Rust & Mitschke GmbH, Bochum, Germany). The processing section of the machine consisted of a single module unit with a total length of 222 mm and an inner diameter of 35.4 mm of the roller cylinder. This was equipped with five standard planetary spindles with a diameter of 8.8 mm and a central spindle with a diameter of 17.7 mm. The length of these was equal to the processing section. Therefore, the overall free processing section volume ($V_{free})$ was 96 mL, while the ratio of heated surface to free process volume was 0.58 m^−1^. An open discharge nozzle with a large diameter for pressure-free release of the granules (Figure 1) was used at the outlet. A gravimetric feeder (DDW-M-DS(R) 28, Brabender GmbH & Co. KG, Duisburg, Germany) was used for dosing of the model formulation, which was blended before processing with a wheel mixer (RRM ELTE 650, J. Engelsmann AG, Lugwigshafen, Germany) for 10 min. Lactose monohydrate (Lactose 310, Foremost Farms USA, Baraboo, WI, USA) served as model compound (90 wt%) and hydroxypropylcellulose (Klucel EXF Pharm, Ashland Inc., Covington, GA, USA) was utilized as melt binder (10 wt%). The set temperature for both heat systems was 150 °C for all experiments. Internal heat exchangers were used with water as tempering media with a volume flow for the central spindle of 7 L min^−1^ and 17 L min^−1^ for the roller cylinder heating. Product temperature was determined on-line with an IR-camera (Test 875, Testo SE & Co. KGaA, Titisee-Neustadt, Germany).

### 2.2. Experimental Design Space

A design space for the execution of the residence time experiments was determined a priori. The set points within the experimental study cover four equidistant levels, each for the investigated process parameter, including the feed rate ($\dot{m}$) from 0.3 to 1.2 $kg\;h^{-1}$ and rotation speed (n) from 60 to 240 ${min}^{-1}$. In combination, these direct process parameters define the specific feed load (SFL), which is a typical characteristic parameter for twin-screw processes. This approach is adapted for planetary roller processing (Equation (3)) and considers the material volume flow, defined by the ratio of feed rate to untapped bulk density ($\rho_{bulk}$), in relation to the potential transport volume flow per one revolution of the central spindle. This correlates to the rotation speed, the pitch of the central spindle ($h_{cs}$), and the free cross-sectional area ($A_{free}$), which in combination with the length of the central spindle ($L_{cs}$) determines the free processing volume ($V_{free}$).

(3) $$SFL=\frac{\dot{m}}{n\;\rho_{bulk}\;h_{cs}\;A_{free}}=\frac{\dot{m}}{n\;\rho_{bulk}\;\frac{h_{cs}}{L_{cs}}\;V_{free}}.$$

### 2.3. Residence Time Characterization

**Figure 3:**
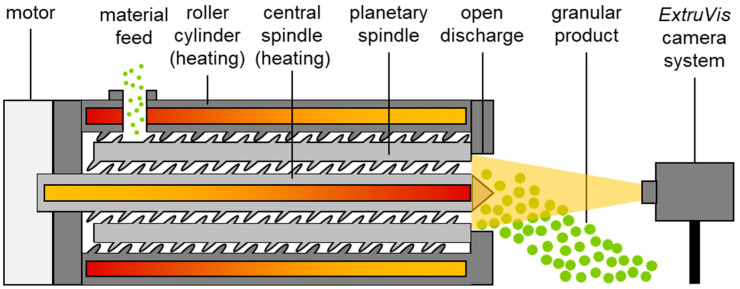
Setup for non-destructive on-line determination of residence time distribution in planetary roller melt granulation experiments via ExtruVis system. Camera sensor placed in front of granulator discharge blend.

Determination of the residence time distribution density function at different process parameter sets was executed with an on-line camera system (ExtruVis, MeltPrep GmbH, Graz, Austria) via pulse signal experiments (Figure 3), which have already been applied successfully to characterize the RTD in twin-screw processes [33,34]. During the steady stage of the process, E104 or Ponceau 4R was added as a tracer to the feed formulation through the hopper, with a ratio of feed rate to tracer mass at 20.83 per second. The corresponding signal was measured at the discharge blend with a frequency of 36 to 51 Hz. To characterize the residence time distribution, a pre-data treatment of the experimental data was executed to derive the E-function. This procedure included a baseline correction as well as a normalization by the overall integral value, which was calculated via the trapezoidal rule. The two-compartment model [35,36] was fitted to the experimental data with the least square method. This model (Equation (4)) considers a material plug flow for the axial transport superimposed by the mixing function of a continuously stirred tank reactor.

(4) $$E\left(t\right)=\frac{k_{0}}{c_{0}}\;0.5\;e^{0.5\;{t_{mix}}^{-3}\sigma^{2}-{t_{mix}}^{-1}\left(t-t_{ds}\right)}\;erfc\left(\frac{{t_{mix}}^{-1}\sigma^{2}-\left(t-t_{ds}\right)}{\sqrt{2}\sigma }\right).$$

The dead time ($t_{ds}$) of the process is the transport time based on the dead process volume in relation to the material flow, whereby the standard deviation (σ) characterizes the axial material distribution of the transport function. For the mixing process, the ratio of the mixing volume to the material volume flow describes the mixing duration ($t_{mix}$) within the reactor and therefore reflects the time extension of the corresponding mechanisms. Furthermore, the initial tracer concentration signal ($c_{0}$) is the ratio of the scaling parameter ($k_{0}$) to the mixing parameter.

## 3. Results and Discussion

### 3.1. Determination and Characterization of the Residence Time

**Figure 4:**
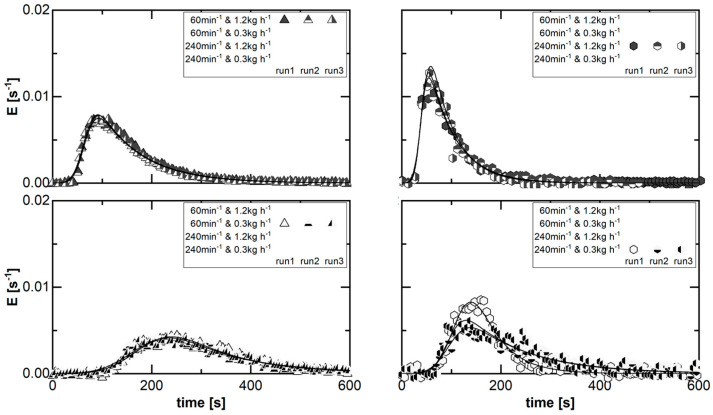
Experimentally determined residence time distribution in steady state at four fixed parameter sets, representing the investigated design space for the investigation of the transport characteristics in planetary roller melt granulation. For the clarity of visual data representation, only every 125th data point is plotted for each data set.

The repeatability concerning the residence time determination was tested at four different set points. These cover the combination of the minimum and maximum levels for each of the adjusted direct process parameters. In steady-state processing, signal impulse experiments were executed in triplicate. The various symbols represent the different runs for each parameter set (Figure 4). The residence time distribution (RTD) is expressed by the density function. At the same time, the suitability of the two-compartment model for the application in planetary roller melt granulation (PRMG) is evaluated by this experimental study. The model fit to the data is displayed in solid lines.

In each case, the characteristic of the RTDs is similar, including a single on-set, peak, and off-set, as well as a prolonged kinetic between peak and off-set in comparison to the kinetic between on-set and peak. This profile matches the common RTD profiles in twin-screw granulation (Figure 2). Furthermore, a sufficient agreement between model fit and experimental data is found for all runs at each fixed parameter set. Therefore, the two-compartment model is classified as suitable for representing the RTD in PRMG.

At the same time, the displayed data also imply a sufficient agreement among the individual repetitions of the residence time determination at a fixed parameter set in a steady state. The visual impression is thereby supported by the fitted model parameters. For the three repetitions, the coefficient of variation (CV) of the individual factors as a ratio of standard deviation to mean value is generally in the range of 10% or less (Table 1). This is sufficient and highlights the repeatability of the RTD determination method.

For the parameter set combining the highest rotation speed at 240 ${min}^{-1}$ and the lowest mass flow at 0.30 $kg\;h^{-1}$, the density of the material flow at the outlet is minimized. This can cause some irregularity in the form of pulsation, as in run 1 (Figure 4, bottom right). The corresponding impact on the RTD measurement and determined profile is limited to the “outwash” kinetic of the RTD. This is the part of the function related to the model mixing parameter ($t_{mix}$). Due to the coupling for the applied RTD model (Equation (4)), $k_{0}$ is also affected. Overall, this is not considered critical, since the described effect has only been observed as an exception for this parameter set in all experimental investigations. However, the potential fluctuation of the model parameters $t_{mix}$ and *k*_0_ for this single parameter set has to be considered during the evaluation of the transport characteristics in PRMG.

### 3.2. Process Parameter Impact on the RTD in PRMG

**Figure 5:**
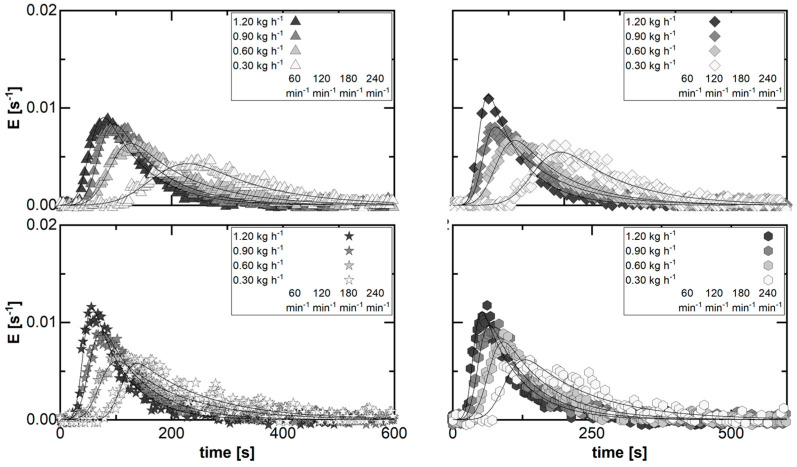
Residence time distribution and model fit to the experimental data for each parameter set of the investigated design space. Granulator configuration, set temperature (central spindle, roller cylinder), and equipment were fixed for all experiments. The symbols equal the applied rotation speed; colour represents applied feed rates. For the clarity of visual data representation, only every 125th data point is plotted for each data set.

The determined RTD profiles for the 16 parameter sets within the design space of the experimental study are given in Figure 5. The data are staggered for the clarity of the depiction in four categories based on a constant rotation speed. The connected model fit is represented by the lines, which are overall in sufficient agreement with the experiments.

For a constant rotation speed, an increase of the feed rate (encoded by the symbol grey scale) generally results in a shift of the peak as well as of the $t_{50}$ (Table 2) to shorter exit times, while the absolute width of the RTD decreases. Therefore, the peak height increases as well. In comparison, increasing the rotation speed (encoded by the symbols) for a constant feed rate has the same effect on the RTD profile. These findings are similar to those in twin-screw processes and the impact of direct process parameter variations on the RTD [37]. Furthermore, the other determined characteristic model parameters (Equation (4)) match the visual findings concerning the process parameter impact.

First of all, the $c_{0}$ values in the range of one indicate a successful normalization of the experimental data to obtain the fundamental density function. The minor deviations are related to the fit of a model, assuming an ideal RTD profile to an experimental data set underlying a method-specific signal-to-noise ratio. In this respect, $k_{0}$ is nearly the reciprocal value of the mixing time.

The dead time represents an exit time for a plug flow in model theory [35], which is not affected by any superimposed material blending or mixing.

Therefore, $t_{ds}$ is connected to the part of the RTD profile before the peak, including the on-set, and decreases for larger feed rates and rotation speeds. The parameter σ is subjected to the same impact, since this represents the absolute width of the exit time distribution of the plug flow function. Instead, the correlations for the mixing time are ambiguous. For a constant rotation speed, the values of $t_{mix}$ decrease with increasing throughput, while an alternating impact is found for an increase of the rotation speed at a constant feed rate. This implies a mixing volume variability, which could originate from an affected material transition pattern between the planetary and central spindle.

### 3.3. Machine Fill Level during PRMG

A key parameter influencing the material transport and mixing during PRMG is the fill level of the granulator during processing. This is defined as the ratio of the processing volume, where the material is actually flowing through to the free processing volume in the roller cylinder, which is not occupied by the central or planetary spindles. For a maximum fill level, the hydrodynamic residence time (τ) represents the average transport due to plug flow. This parameter (Equation (5)) correlates to the granulator load.

(5) $$\tau =\frac{V_{free}}{\;\dot{V}\;}=\frac{V_{free}}{\;\frac{\dot{m}}{\rho_{bulk}}\;\;}=\frac{1}{\;n\;\frac{h_{cs}}{L_{cs}}\;SFL\;\;}.$$

**Figure 6:**
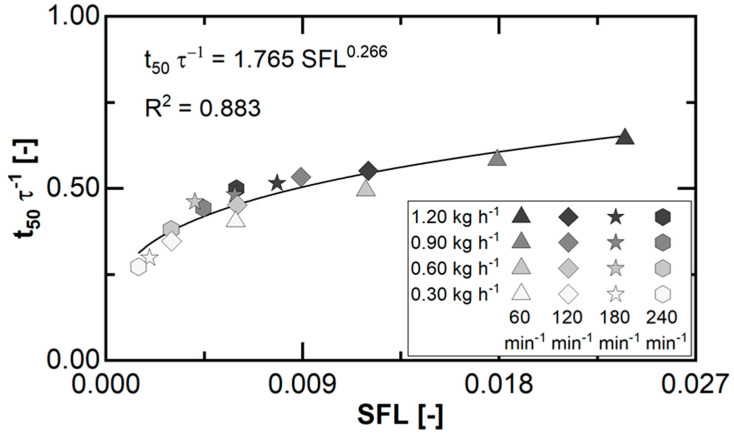
Ratio of RTD median to hydrodynamic residence time as surrogate for the granulator fill level parameter for each parameter set of the investigated design space (Figure 3). Granulator configuration, set temperature (central spindle, roller cylinder), and equipment were fixed for all experiments. The symbols equal the applied rotation speed, colour represents applied feed rates. Line represents the fit of a power model approach to the overall displayed data.

For the overall PRMG process, the median of the RTD represents the average axial transport time based on the covered free processing volume within the machine by the material stream. Therefore, the ratio of $t_{50}$ to τ can be applied as a surrogate for the granulator fill level (Figure 6).

In general, the surrogate for the fill level is constantly below 100% (Table 2) and increases for a higher SFL, and the corresponding correlation fits a power model approach. Therefore, the impact of the investigated process parameter variations and the fill level adjustment in steady state depends on the balance of the acting forces related to the material transport in PRMG.

On the one hand, this includes the applied specific mechanical energy, which is transmitted at the contact surface of planetary spindles and central spindle, respectively, and the roller cylinder. Due to the 45° toothing and the orbital motion of the planetary spindles, the transferred force ($F_{transfer}$) is converted into axial drive and radial distribution of the bulk material. Thus, the material is sheared by the moving mechanical parts. Consequently, the overall transferred forces rely on the shear rate depending on the rotation speed, viscosity (η), and the covered contact surface. This must directly correlate to the covered cross-sectional area by the material stream ($A_{free,cov})$, which represents the granulator fill level. On the other hand, the axial drive of the material resulting in a transport velocity is limited due to friction and dissipation. In fluid dynamics, this is usually referred to as pressure loss. The corresponding force ($F_{friction}$) depends on the feed rate ratio in the power of two to the bulk density and the covered cross-sectional area by the material stream, which represents the granulator fill level.

The overall force balance for the material transport can be summarized (Equation (6)). However, the material-related parameters can be neglected for the conducted experimental investigations, as the material formulation was constant.

(6) $$F_{transferred}\left(n,\;\eta ,\;A_{free,cov}\right)=F_{friction}\left({\dot{m}}^{2},\;{\rho_{bulk}}^{-1},\;{A_{free,cov}}^{-1}\right).$$

For a constant rotation speed, a higher SFL refers to a higher feed rate. The corresponding amplifying impact on the friction force is balanced by increasing the flow through the cross-sectional area. This reduces the flow velocity and, thereby, the friction force and enhances the transferred energy. For a constant feed rate, a higher SFL refers to a lower rotation speed. The corresponding attenuating impact on the transferred force is balanced by increasing the flow through the cross-sectional area. This reduces the friction force, even while the throughput is maintained.

However, the efficiency of the mechanical energy transfer decreases with higher fill levels since the material is initially distributed in the contact area of the mechanical parts. Later on, the material fills up the void spaces between the planetary spindles, which barely contribute to the energy input. Furthermore, based on the determined particle size distributions of the granules in one study [21], a higher fill level contributes to the mechanisms of nucleation and agglomeration [29] and leads to an enlarged particle size.

### 3.4. Material Transport and Mixing Characteristics in PRMG

**Figure 7:**
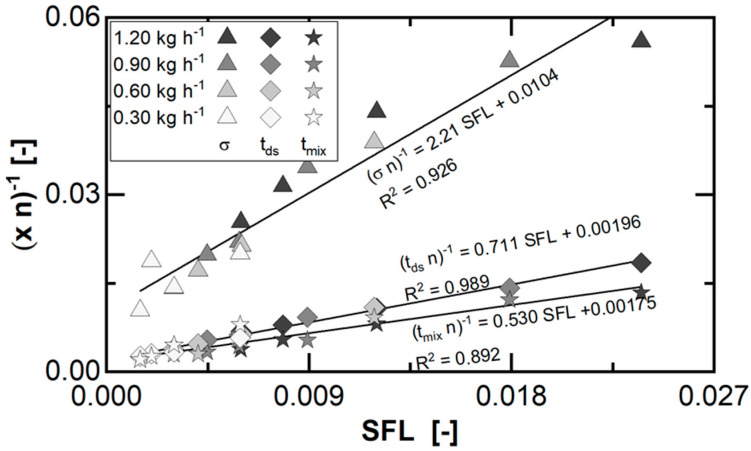
Fitted and transformed transport and mixing-related parameters of the applied two-compartment model to the experimental data for each parameter set of the investigated design space. Granulator configuration, set temperature (central spindle, roller cylinder), and equipment were fixed for all experiments. The symbols equal the model parameter, colour represents applied feed rates. Rotation speeds decrease for higher SFL at a constant feed rate. Lines represent the fit of a linear model approach to the overall displayed data.

With respect to the applied RTD model, the axial material transport is characterized by $t_{ds}$, while axial back-mixing is related to σ and radial material distribution by $t_{mix}$. Due to the machine concept, including the 45° toothing of the mechanical parts as well as the orbital motion of the planetary spindles around the central spindle, all mentioned parameters and the corresponding mechanisms are coupled. Moreover, these are related concerning the model theory to the granulator fill level, which correlates to the SFL (Figure 6). In this context, a transformation of the time parameters (Equation (5)) is considered to display the data (Figure 7).

In general, all transformed parameters correlate linearly to the SFL, which justifies the applied parameter transformation. The values for σ are the highest at each set point ((*σ n*)^−1^ = 2.21 SFL + 0.0104), as the width of the axial material transport is absolutely lower than the duration of the material transport (Table 2). Furthermore, transport and mixing time are in the same order of magnitude ((*t_ds_ n*)^−1^ = 0.711 SFL + 0.00196, (*t_mix_ n*)^−1^ = 0.530 SFL + 0.00175), since the corresponding mechanisms refer to the same fill volume inside the granulator. The higher values for $t_{mix}$ result from the superimposition of the orbital planetary spindle movement and their self-rotation on the radial distribution by the part toothing. This leads to an apparent increase in the mixing volume in comparison, due to a circulation within and exchange in between distinct material portions continuously during processing along the granulator.

Furthermore, the impact of the process parameter adjustments on the transformed model parameters is directly related to the granulator fill level. This is also manipulated by a variation of the feed rate or the rotation speed (Figure 7), while the other one is constant. However, the relative factor a parameter adaption leading to a fill level increase is exceeding the relative factor of the corresponding fill volume increase. Therefore, the transformed surrogates decrease while, in turn, the model parameters representing a duration as ratio of a volume to feed rate increase. This effect is due to the balance regarding the transferred shear forces by the mechanical parts to the material and the friction force limiting the material discharge (Equation (6)). Here, the fill level acts as an equalizer having a multiple influence on the acting forces. Furthermore, the manipulation of the mechanical energy transferred into the material is less effective with an increasing load, as the material in the void space is barely taking part in the interaction in the contact area of the mechanical parts. At the same time, the mixing volume is related to the fill volume as well as the number of material circulations. These are driven by the self-rotation of the planetary spindles and the transition patterns between planetary and central spindles. Therefore, a reduction in the rotation speed has an increasing effect on the fill level, while the reduced circulation of the fill volume has a decreasing effect on the mixing volume. This adds to the limited effectiveness of the parameter adaption to manipulate the fill level.

## 4. Conclusions

The residence time distribution is a key indicator for the processing conditions during planetary roller melt granulation, as this indirect process parameter reflects the material transport. This especially includes the actual fill level, which represents the covered fraction of the free processing volume by the material during processing. The free volume in a single module is determined by the type and number of planetary spindles considered in a configuration. During processing, the fill level itself is adjustable by the direct process parameters, which simultaneously affect the axial material transport and the radial material distribution. These, in turn, determine the transport velocity of the material and the mixing time. Both aspects contribute to the granulation performance during planetary roller melt granulation. However, in the common setup, which was also used for these experimental investigations, the pre-mix of binder and model component resulted in a superimposition of granulation and melting during processing. Therefore, the residence time distribution reflects both mechanisms and cannot be directly correlated to the product particle size distribution.

## Figures and Tables

**Table 1:** Coefficient of variation (CV) of the determined RTD model parameters for the three repetitions at the different process parameter set points defined by the set rotation speed and feed rate.

*n_set_* [min^−1^]	${\dot{m}}_{\mathrm{set}}$ [kg h^−1^]	${CV}_{k_{0}}$ [%]	${CV}_{t_{ds}}$ [%]	${CV}_{\sigma }$ [%]	${CV}_{t_{mix}}$ [%]
60	0.3	11.1	3.28	7.83	10.1
60	1.2	2.59	1.34	3.69	2.44
240	0.3	47.9	8.81	11.0	41.3
240	1.2	10.4	3.46	11.6	6.49

**Table 2:** Parameter matrix for the determined RTD model fits to the experimental data.

*n_set_* [min^−1^]	${\dot{m}}_{\mathrm{set}}$ [kg h^−1^]	SFL [−]	$t_{50}$ [s]	$c_{0}$ [−]	$k_{o}$ [−]	$t_{ds}$ [s]	*σ* [s]	$t_{mix}$ [s]	$t_{50}\tau^{-1}$ [%]
60	0.3	0.00595	269.9	1.01	0.00805	172.4	49.6	124.8	40.4
60	0.6	0.00300	165.2	1.06	0.00977	91.4	25.6	108.4	34.6
60	0.9	0.00200	129.7	1.05	0.01282	70.4	19.0	81.9	29.8
60	1.2	0.00149	107.7	1.04	0.01396	53.9	17.8	74.2	27.1
120	0.3	0.00191	231.0	1.03	0.00937	149.5	35.0	110.4	49.4
120	0.6	0.00601	151.2	0.98	0.01046	79.1	23.5	94.2	45.2
120	0.9	0.00408	118.8	1.02	0.01099	54.1	14.4	92.8	46.1
120	1.2	0.00299	92.1	1.00	0.01620	46.6	11.4	61.9	38.1
180	0.3	0.01791	199.3	0.97	0.00774	109.4	17.8	125.5	58.2
180	0.6	0.00894	154.2	0.96	0.00849	71.7	19.9	113.5	53.3
180	0.9	0.00591	107.4	1.00	0.01330	52.7	15.0	75.6	48.2
180	1.2	0.00447	86.1	0.97	0.01612	41.4	10.4	60.3	44.3
240	0.3	0.02377	181.3	1.08	0.00902	94.5	24.0	120.3	64.5
240	0.6	0.01201	127.3	1.00	0.01189	65.2	17.7	84.5	55.1
240	0.9	0.00783	98.6	1.00	0.01362	46.2	12.6	73.5	51.5
240	1.2	0.00597	83.5	0.98	0.01500	36.6	9.8	65.6	50.0

## Data Availability

The data presented in this study are available on request from the corresponding author.

## Nomenclature

$A_{free}$	Free area of the planetary roller processing cross-section	[m^2^]
$A_{free,cov}$	Free area of the planetary roller processing cross-section covered with material during processing	[m^2^]
$c_{0}$	Signal strength of initial tracer concentration	[*]
*CV*	Coefficient of variation	[-]
E	Residence time density function	[s^−1^]
*η*	Dynamic viscosity	[Pa s]
F	Cumulative residence time function	[-]
$F_{friction\;}$	Friction force	[N]
$F_{transferred}$	Transferred force via rotating parts	[N]
$h_{cs}$	Pitch of central spindle	[mm]
$k_{0}$	Scaling parameter	[s^−1^]
$l_{cs}$	Length of central spindle	[mm]
$\dot{m}$	Feed rate of material pre-mix	[kg h^−1^]
*n*	Rotation speed of central spindle	[min^−1^]
$\rho_{bulk}$	Untapped bulk density	[kg m^−3^]
*SFL*	Specific feed load	[-]
*σ*	Transport function width	[s]
*t*	Time	[s]
$t_{50}$	Median of residence time distribution	[s]
$t_{dead}$	Process dead time	[s]
$t_{mix}$	Process mixing time	[s]
*τ*	Hydrodynamic mean residence time	[s]
$\dot{V}$	Process volume flow	[m^3^ h^−1^]
$V_{free}$	Free volume of the planetary roller processing section	[m^3^]
